# Case Report: Successful Treatment With Anti-C5 Monoclonal Antibody in a Japanese Adolescent Who Developed Thrombotic Microangiopathy After Autologous Bone Marrow Transplantation for Malignant Lymphoma

**DOI:** 10.3389/fped.2022.908183

**Published:** 2022-07-04

**Authors:** Shoichi Shimizu, Tamaki Morohashi, Koji Kanezawa, Hiroshi Yagasaki, Shori Takahashi, Ichiro Morioka

**Affiliations:** ^1^Department of Pediatrics and Child Health, Nihon University School of Medicine, Tokyo, Japan; ^2^Itabashi Central Medical Center, Tokyo, Japan

**Keywords:** transplant-associated thrombotic microangiopathy (TA-TMA), anti-C5 monoclonal antibody (eculizumab), membrane attack complex (C5b-9), acute kidney injury (AKI), graft-versus-host disease (GVHD)

## Abstract

**Background:**

Transplant-associated thrombotic microangiopathy (TA-TMA) is a serious complication of bone marrow transplantation (BMT). Recently, abnormalities in the complement system have been identified in the pathogenesis of TA-TMA, and there are series of reports stating that anti-C5 monoclonal antibody (eculizumab) is effective in patients with high levels of the membrane attack complex (C5b-9).

**Case Presentation:**

A 12-year-old boy underwent autologous BMT after receiving high-dose chemotherapy for malignant lymphoma. The patient was engrafted on day 19 after transplantation; however, hemolytic anemia and non-immune thrombocytopenia persisted, and haptoglobin decreased on day 46. Moreover, on day 83, the patient developed pulmonary hemorrhage, hypertension, severe proteinuria, hematuria, and acute kidney injury (AKI). Pulmonary bleeding stopped with daily platelet transfusion and hemostatic agents, but reappeared on day 100. Based on the presence of destruction of red blood cells, elevated lactate dehydrogenase levels, negative direct and indirect Coombs tests, normal ADAMTS13 levels, hemolytic anemia, non-immune thrombocytopenia, and AKI, the patient was diagnosed with systemic TA-TMA and we initiated plasma exchange (PE) and continuous hemodialysis for AKI. High C5b-9 levels were identified at the start of the series of PE, therefore we decided to administer eculizumab. After three courses of eculizumab, no pulmonary hemorrhage was observed, and anemia, thrombocytopenia, renal dysfunction, hematuria, and proteinuria all tended to improve. Three years after transplantation, the patient is alive and does not require eculizumab.

**Discussion:**

Eculizumab is a humanized monoclonal antibody that binds complement protein C5, preventing cleavage C5 and the formation of C5b-9. In this case, TA-TMA could not be controlled with PE alone. We therefore decided to use eculizumab relatively early based on the high C5b-9 level and could resolve the momentum of TA-TMA.

**Conclusion:**

In previous reports, TA-TMA typically occurred in early post-allogeneic BMT of patients with lymphoma or in post-autologous BMT of patients with neuroblastoma and was treated with eculizumab. We here reported that eculizumab could be successful treatment for TA-TMA in post-autologous BMT of patient with lymphoma.

## Introduction

Transplant-associated thrombotic microangiopathy (TA-TMA) is an increasingly recognized complication of bone marrow transplantation (BMT) with high rates of morbidity and mortality. TA-TMA is characterized by microangiopathic hemolytic anemia, consumptive thrombocytopenia, and organ damage due to microcirculatory failure (1) and is associated with approximately 10–25% of allogeneic transplants (2).

The clinical findings of TA-TMA include rapid progression of anemia, fragmentation of red blood cells, delayed platelet recovery, transfusion refractoriness, elevated serum lactate dehydrogenase (LDH) levels, and acute renal failure. Disease onset often occurs after leukocyte engraftment and up to 100 days after transplantation (3).

Although the pathophysiology of TA-TMA is not fully understood, it is ultimately caused by vascular endothelial cell damage in multiple organs, including the kidney, lung, intestine, and central nervous system (4). The causes of TA-TMA include intense chemotherapy and total body irradiation during pre-transplant treatment, administration of calcineurin inhibitors for graft-versus-host disease (GVHD) prophylaxis, complications of severe GVHD, and post-transplant infections (5, 6).

TA-TMA is a poor prognostic complication of BMT, and even if the patient survives, irreversible damage to the renal tissue may occur, leading to chronic kidney disease (CKD) in some cases (7).

There is currently no standard of care for TA-TMA, and mortality is high, despite the use of plasma exchange (PE). Eculizumab, an anti-C5 monoclonal antibody, inhibits the formation of the terminal membrane attack complex (C5b-9) and TMA progression (8, 9). Eculizumab is commonly used as a treatment for paroxysmal nocturnal hemoglobinuria (PNH) and atypical hemolytic uremic syndrome (HUS), and there are a series of reports that were also effective in patients with TA-TMA which has similar presentation and pathophysiology to atypical HUS (10–12).

Previous reports have shown that TA-TMA usually occurred in early post-allogeneic BMT of patients with lymphoma or in post-autologous BMT of patients with neuroblastoma, and the therapeutic effects of eculizumab have been recognized in these pathological conditions (11, 13). However, there are few reports of the use of eculizumab for TA-TMA that occurs in post-autologous BMT of patient with lymphoma. Herein, we report the case of a 12-year-old boy who underwent autologous BMT after high-dose chemotherapy for primary gastric lymphoma and developed TA-TMA that was successfully treated with eculizumab.

## Case Description

A 12-year-old patient underwent autologous BMT after high-dose chemotherapy included etoposide, carboplatin, and cyclophosphamide for malignant lymphoma. The patient was engrafted on day 19 after transplantation; however, anemia and non-immune thrombocytopenia persisted. Additionally, the bone marrow findings on day 31 showed hypoplastic marrow, and haptoglobin levels suggestive of the presence of hemolytic anemia decreased to 15 mg/dL on day 46. He had hypertension due to intravascular overflow and needed a calcium blocker. Bloody sputum and respiratory distress appeared on day 83, and chest computed tomography showed diffuse pulmonary hemorrhage and bilateral pleural effusions. Although pulmonary bleeding stopped with platelet transfusion and hemostatic agents, it reappeared on day 100. Furthermore, the patient developed hypertension, severe proteinuria, and hematuria. Laboratory tests showed anemia associated with fragmented red blood cells and elevated LDH levels consistent with hemolytic anemia, negative direct and indirect Coombs tests, normal ADAMTS13 levels, and non-immune thrombocytopenia. Acute kidney injury (AKI) was defined as elevated creatinine (Cr) levels and decreased urine output (Table 1).

**Table 1:** Blood and urine test findings at the time of the second pulmonary hemorrhage (Parentheses indicate normal values).

**Blood**					
WBC	3,400 (3,300~8,600)	/μL	**T-Bil**	**3.47** (0.4~1.5)	**mg/dL**
Neu	81.0	%	**D-Bil**	**0.84** (0.05~0.4)	**mg/dL**
Mono	6.0	%	AST	39 (13~30)	IU/L
Lymph	13.0	%	ALT	55 (10~42)	IU/L
**RBC**	**2.15** **×10**^**6**^ (4.35~5.55 ×10^6^)	**/μL**	**LDH**	**883** (124~222)	**IU/L**
**Hb**	**6.2** (13.7~16.8)	**g/dL**	**BUN**	**47.1** (8~20)	**mg/dL**
**Ht**	**19.2** (40.7~50.1)	**%**	**Cr**	**1.21**	**mg/dL**
**Plt**	**3.2** **×10**^**4**^ (15.8~34.8 ×10^4^)	**/μL**	Na	146 (138~145)	mmol/L
**Ret**	**45** (4~20)	‰	K	3.3 (3.6~4.8)	mmol/L
			Cl	112 (101~108)	mmol/L
			CRP	0.40 (<0.2)	mg/dL
			TP	5.9 (6.6~8.1)	g/dL
			Alb	3.5 (4.1~5.1)	g/dL
			**Cr-eGFR**	**63.9** (>90)	**mL/min/ 1.73m2**
**Blood**			**Urine**		
PT ratio	1.01 (0.9~1.1)		pH	6.5 (5.0~7.0)	
APTT	39.9 (27~45)	sec	SG	1.023 (1.005~1.02)	
Fib	293 (150~400)	mg/dL	**Protein**	**(4+)**	
AT-3	80 (70~130)	%	**Sugar**	**(1+)**	
**FDP**	**12.0** (<5)	**μg/dL**	**OB**	**(3+)**	
**D-dimer**	**5.4** (<5)	**μg/dL**	URO	(±)	
			Bil	(-)	
**Cystatin C**	**1.08** (0.58~0.98)	**mg/L**	Ket	(-)	
Renin	0.6 (0.2~3.9)	ng/mL/hr	**RBC**	**50** **~** **99**	**/HPF**
Aldosterone	10.0 (<173)	pg/mL			
			**NAG**	**48.6** (<5)	**IU/L**
**CH50**	**50.5** (30~45)	**U/mL**	**β2MG**	**10048** (5~300)	**μg/L**
**Haptoglobin**	**<10** (19~170)	**mg/dL**			
ADAMTS13	92 (50~150)	%	**U-TP/Cre**	**23.05** (<0.2)	**g/ gCr**
direct coombs	(-)				
indirect coombs	(-)				

*Blood and urine showed fragmented red blood cells, elevated LDH and creatinine levels, negative direct and indirect Coombs tests, normal ADAMTS13 levels, hemolytic anemia, and non-immune thrombocytopenia*.

*WBC, white blood cell; Neu, neutrophils; Mono, monocyte; Lymph, lymphocytes; RBC, red blood cell; Hb, hemoglobin; Ht, hematocrit; Plt, blood platelet; Ret, reticulocytes; pH, pounds hydrogenii; PaCO_2_, alveolar carbon dioxide tension; PaO_2_, partial pressure of arterial oxygen; HCO^3−^, bicarbonate ion; BE, base excess; T-Bil, total bilirubin; D-Bil, direct bilirubin; AST, aspartate aminotransferase; ALT, alanine aminotransferase; LDH, lactate dehydrogenase; BUN, urea nitrogen; Cr, creatinine; Na, sodium; K, potassium; Cl, chloride; CRP, C-reactive protein; TP, total protein; Ab, albumin; Cr-eGFR, creatinine-estimated glomerular filtration rate; PT, prothrombin time; APTT, activated partial thromboplastin time; Fib, fibrinogen; AT-3, antithrombin; FDP, fibrin degradation product; CH50, 50% hemolytic unit of complement; ADAMSTS13, a disintegrin-like metalloproteinase with thrombospondin type 1 motifs 13; SG, specific gravity; OB, occult blood; URO, urobilinogen; UTP/Cr, Urinary protein-creatinine ratio; Bil, bilirubin; Ket, ketone; NAG, N-acetylglucosaminidase; β2MG, β2 -microglobulin; U-TP/Cre, urine protein-to-creatinine ratio. The bold indicates out of normal range*.

The patient was finally diagnosed as having systemic TA-TMA based on the diagnostic criteria of Jodele et al. (9), and we initiated daily PE along with continuous hemodialysis for AKI. Despite a decrease in Cr and an upward trend in platelet level, pulmonary hemorrhage could not be controlled, leading to temporary endotracheal intubation and artificial respiration management to secure the airway and positive pressure ventilation. However, the therapeutic effect of PE on TA-TMA was limited. Because of the high C5b-9 levels (213.9 ng/ml, normal 85.5 ± 21.1 ng/ml) at the beginning of PE, we decided to stop PE and to administer eculizumab. Eculizumab was administered once per week for a total of three doses. We prescribed eculizumab after receiving approval for its off-label use in the hospital. We also obtained full informed consent from the patient and his parents, and the patient was administered a meningococcal vaccine with eculizumab. The patient was also given cefozopran hydrochloride as antimicrobial prophylaxis adequate to cover meningococcal infection.

**Figure 1:**
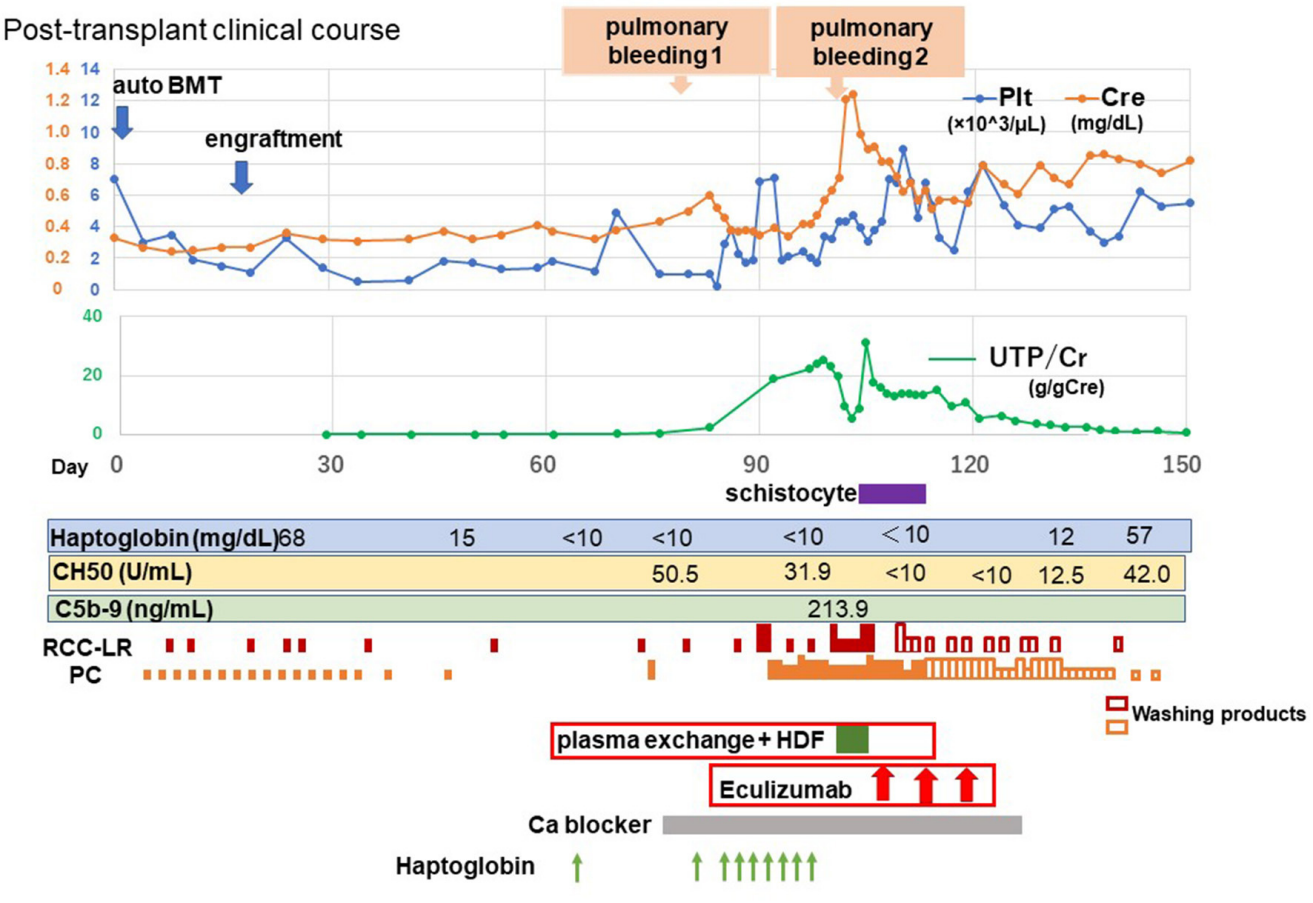
Post-transplant clinical course. Platelet count, serum creatinine level, urine protein-creatinine ratio, haptoglobin, and complement titers are also shown to illustrate changes in transplant-associated thrombotic microangiopathy disease status. BMT, Bone marrow transplantation; Plt, blood platelet; Cr, creatinine; U-TP/Cre, urine protein-to-creatinine ratio; CH50, 50% hemolytic unit of complement; C5b-9, membrane attack complex; HDF, hemodiafiltration; RCC-LR, red cell concentrates-leukocytes reduced; PC, platelet concentrate; Ca, calcium.

Jodele et al. proposed an algorithm for effective administration of eculizumab to patients with TMA using CH50 levels and serum levels of eculizumab (14). When steady CH50 suppression is achieved and hematologic TMA parameters and plasma sC5b-9 normalize, eculizumab should be advanced to a maintenance schedule. Then, if TMA remains controlled after 3 to 4 maintenance doses, eculizumab may be discontinued. In this case, after three courses of eculizumab, no pulmonary hemorrhage was observed, and hemolysis, thrombocytopenia, renal dysfunction, hematuria, and proteinuria tended to improve. Thereafter, the complement levels normalized, and regular eculizumab infusion was not necessary. Two months later, the patient became transfusion independent (Figure 1).

**Figure 2:**
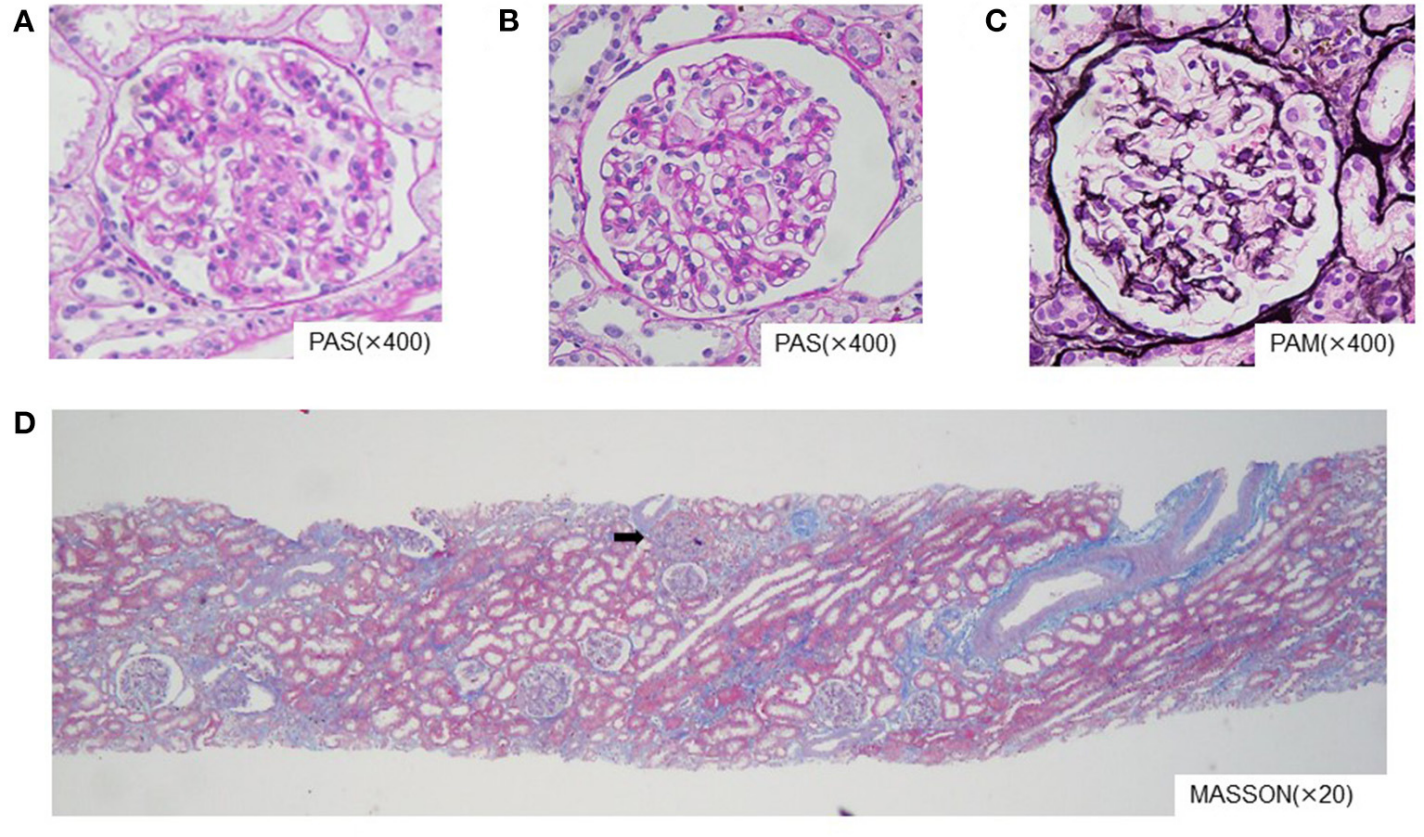
Pathological findings (optical microscopy). **(A,B)** Two different glomeruli at 400x of PAS. **(C)** Glomerulus at 400x of PAM. **(D)** Kidney tissue at 20x of MASSON. The changes in the basement membrane of the hoof were minimal, mesangial proliferation was partial, and fibrosis was observed in only 10% of the cases. No membranoproliferative glomerulonephritis-like findings were observed in the chronic phase of impaired TMA, and there were only two sclerotic glomeruli (arrow) out of 18. PAS, periodic acid Schiff stain; PAM, periodic acid-methenamine-silver stain; MASSON, masson trichrome stain.

Ten months after TA-TMA onset, renal biopsy was performed to evaluate the long-term prognosis of the kidney. The estimated glomerular filtration rate (eGFR) of Cr was 119.7 mL/min/1.73 m2 at the time of the biopsy. Changes in the basement membrane of the glomeruli were minimal, mesangial proliferation was partial, and fibrosis was only observed in 10% of the entire tissue. There were only two sclerotic glomeruli, out of 18 (Figure 2). The immunofluorescence antibody method showed focal and segmental staining of IgM and fibrinogen, which was consistent with TMA, and there were no obvious abnormal electron microscopic findings. The membranoproliferative glomerulonephritis-like changes in glomeruli, global glomerular sclerosis, arterial fibrous thickening, tubular atrophy, and interstitial fibrosis that were observed during the chronic repair phase of TMA were absent. From these findings, we presume that the damage to the renal tissue caused by TMA was resolved early and the tissue repair was good. Three years have passed since the onset of TA-TMA, and the current eGFR of Cr is 142 mL/min/1.73 m^2^, maintaining a normal level.

## Discussion

Currently, no effective treatment for TA-TMA has been established. Treatment of comorbidities and reduction or discontinuation of calcineurin inhibitors cannot be deemed as a positive treatment for progressive TMA.

PE is effective in diseases such as acquired thrombotic thrombocytopenic purpura, in which ADAMTS13 activity is severely reduced. Additionally, it has been reported to be effective against TA-TMA (15). However, in TA-TMA, the effect of PE is poor in terms of both survival and renal function (16). In particular, diffuse alveolar hemorrhage has been reported to be a potentially fatal complication that occurs at a high rate in patients with TA-TMA (17). Further, high proteinuria and high C5b-9 levels, as in this case, are poor prognostic factors for TA-TMA (1) and require more aggressive treatment.

Eculizumab is a humanized monoclonal antibody to complement protein C5 that can prevent tissue damage by inhibiting the formation of C5b-9 as a treatment for PNH and atypical hemolytic uremic syndrome (18). The mechanism of action against TA-TMA is thought to prevent tissue damage by inhibiting the production of C5b-9. In Japan, eculizumab is not approved for the treatment of TA-TMA; nevertheless, other investigators have recently reported that eculizumab is useful for the treatment of TA-TMA (8, 9). Jodele et al. reported that 18 patients with TA-TMA with high proteinuria and high C5b-9 levels were managed with eculizumab and 11 without, and the 1-year survival rates were 56 and 9%, respectively (13). Atypical hemolytic uremic syndrome (aHUS) is caused by uncontrolled activation of the alternative complement pathway, which results in TMA. In the treatment of aHUS with TMA, there was a difference in the recovery of eGFR between patients who received eculizumab within 1 week after the onset and those who received it after 1 week (14); the mean eGFR change from baseline at 1 year was significantly higher in patients treated for ≤ 7 days than in those treated for >7 days. This indicates that eculizumab has been shown to be more effective in TMA when started early.

The significance of eculizumab in this case is as follows. First, C5b-9 levels were measured in early stage of TMA, immediately before PE, and used for eculizumab induction, which allowed the affected child to survive without complications. Renal function was reversible particularly, and renal biopsy revealed very little irreversible tissue damage. The patient's renal function remained normal even after several years. Second, eculizumab was administered only thrice, making it less expensive. Additionally, it is very interesting that this patient required to take eculizumab only three times, because most of the published literature shows that a prolonged eculizumab course should be needed. Shorter courses of eculizumab may be possible if the drug is started early and the drug monitoring is appropriately performed. Finally, several years have passed since the onset of TA-TMA without relapse.

In Japan, there was a case report which stated that eculizumab was useful for TA-TMA, and the rationale for its use is the same as in other countries with high C5b-9 levels (19). In addition, it has been proposed that proteinuria plus C5b-9 can be used as a diagnostic criterion for TA-TMA in other countries (9).

In this case, complement (C) 4d in the renal tissue, especially the glomeruli and renal tubules, was not studied. In patients with TA-TMA, C4d deposition in the renal tissue may reflect complement activity, and therefore, if evaluated, could have been used to assess disease activity at the time of renal biopsy.

## Conclusion

Autologous stem cell transplantation with high-dose chemotherapy in patients with neuroblastoma has been reported to be a high risk for TA-TMA (20). Herein we reported a case of TA-TMA after autologous stem cell transplantation in a patient with lymphoma and successful treatment with eculizumab. The use of eculizumab improved the condition of TA-TMA and showed functional and pathological reversibility in the kidney. It could be valuable to use eculizumab in the early phase of TA-TMA using C5b-9 levels as one of the rationales, unless other life-saving measures are available.

## Data Availability Statement

The original contributions presented in the study are included in the article/supplementary material, further inquiries can be directed to the corresponding author/s.

## Author Contributions

SS carried out the assessment and the management of the patient, literature search, and drafted the manuscript. TM evaluated histopathological features, contributed histological part, and supported drafting of manuscript. HY supported the evaluation and management of the patient and drafting of manuscripts. KK contributed to the management of the patient. ST and IM reviewed and revised the manuscript for important intellectual content. All authors read and approved the final manuscript.

## Conflict of Interest

The authors declare that the research was conducted in the absence of any commercial or financial relationships that could be construed as a potential conflict of interest.

## Publisher's Note

All claims expressed in this article are solely those of the authors and do not necessarily represent those of their affiliated organizations, or those of the publisher, the editors and the reviewers. Any product that may be evaluated in this article, or claim that may be made by its manufacturer, is not guaranteed or endorsed by the publisher.
